# Seroprevalence of antibodies to primate erythroparvovirus 1 (B19V) in Australia

**DOI:** 10.1186/s12879-018-3525-7

**Published:** 2018-12-07

**Authors:** Helen M. Faddy, Elise C. Gorman, Veronica C. Hoad, Francesca D. Frentiu, Sarah Tozer, R. L. P. Flower

**Affiliations:** 1 Research and Development, Australian Red Cross Blood Service, Brisbane, Queensland Australia; 2 School of Biomedical Sciences, and Institute for Health and Biomedical Innovation, Queensland University of Technology, Brisbane, Queensland Australia; 3 Clinical Services and Research, Australian Red Cross Blood Service, Perth, Western Australia Australia; 4 Queensland Paediatric Infectious Diseases Laboratory, Centre for Children’s Health Research, Brisbane, Queensland Australia

**Keywords:** Seroprevalence, Parvovirus B19, Public health, Paediatric, Australia

## Abstract

**Backgroud:**

Primate erythroparvovirus 1 (B19V) is a globally ubiquitous DNA virus. Infection results in a variety of clinical presentations including erythema infectiosum in children and arthralgia in adults. There is limited understanding of the seroprevalence of B19V antibodies in the Australian population and therefore of population-wide immunity. This study aimed to investigate the seroprevalence of B19V antibodies in an Australian blood donor cohort, along with a cohort from a paediatric population.

**Methods:**

Age/sex/geographical location stratified plasma samples (*n* = 2221) were collected from Australian blood donors. Samples were also sourced from paediatric patients (*n* = 223) in Queensland. All samples were screened for B19V IgG using an indirect- enzyme-linked immunosorbent assay.

**Results:**

Overall, 57.90% (95% CI: 55.94%–59.85%) of samples tested positive for B19V IgG, with the national age-standardized seroprevalence of B19V exposure in Australians aged 0 to 79 years estimated to be 54.41%. Increasing age (*p* < 0.001) and state of residence (p < 0.001) were independently associated with B19V exposure in blood donors, with the highest rates in donors from Tasmania (71.88%, 95% CI: 66.95%–76.80%) and donors aged 65–80 years (78.41%, 95% CI: 74.11%–82.71%). A seroprevalence of 52.04% (95% CI: 47.92%–56.15%) was reported in women of child-bearing age (16 to 44 years). Sex was not associated with exposure in blood donors (*p* = 0.547) or in children (*p* = 0.261) screened in this study.

**Conclusions:**

This study highlights a clear association between B19V exposure and increasing age, with over half of the Australian population likely to be immune to this virus. Differences in seroprevalence were also observed in donors residing in different states, with a higher prevalence reported in those from the southern states. The finding is consistent with previous studies, with higher rates observed in countries with a higher latitude. This study provides much needed insight into the prevalence of B19V exposure in the Australian population, which has implications for public health as well as transfusion and transplantation safety in Australia.

## Background

Primate erythroparvovirus 1, known as parvovirus B19 or B19 virus (B19V), is a globally ubiquitous DNA virus that may occur more frequently in temperate rather than tropical countries [1, 2]. B19V is a small, non-enveloped, single stranded DNA virus, belonging to the *Parvoviridae* family [3, 4]. Three genotypes (genotype 1–3) exist, with genotype 1 the most prevalent world-wide [5]. B19V has a tropism towards human erythroid cells, with the P-blood group antigen serving as the cellular receptor [6]. B19V replicates in bone marrow in erythroid colony forming units, erythroid burst forming units and erythroid precursor cells and has been detected in foetal cardiac, liver and placental cells [7, 8]. The α5β1 integrin complex has also been defined as a co-receptor for the entry of B19V into permissive cells, such as erythroid progenitor cells and other non-erythroidal cells [9]. Transmission is usually through the respiratory route, however, vertical transmission, transmission through solid organ or haematopoietic transplantation, and transfusion-transmission have also been documented [10–12].

Approximately 25% of infected healthy individuals are asymptomatic [5]. Where symptoms are observed, the most common clinical manifestation in paediatric patients is erythema infectiosum, commonly known as fifth disease or slapped cheek syndrome [13]. B19V has also been shown to play a role in the aetiology of severe anaemia in paediatric patients [14]. Clinical manifestations are observed approximately one week after initial exposure in healthy adults, and can include influenza-like symptoms, rash, polyarthralgia, myalgia, and acute-onset oligoarthritis [15–19]. Vertical transmission of B19V from mother to foetus has been documented, with adverse manifestations including hydrops fetalis, where fluid accumulates in foetal compartments causing complications or death [20]. Clinical manifestations of B19V in immunocompromised patients, chronic anaemia, and patients undergoing chemotherapy, are generally atypical [21]. They present with persistent to severe anaemia, fever, lacy skin rash, arthropathy, cardiomyopathy, transient aplastic crisis, and pancytopenia [21]. Transient aplastic crises occurs in those with erythrocyte diseases for example sickle cell disease, thalassaemia and spherocytosis [22]. In those with sickle cell disease it can be life-threatening without prompt treatment [22]. Neurological manifestations, such as encephalopathy and encephalitis, have also been associated with B19V infection [23]. B19V also has the ability to reactivate in immunocompromised patients, which may create difficulties in differentiating between transfusion-transmission and reactivation [24]. The role of B19V in other disease aetiologies, such as other hemotological syndromes, is not well established and there is uncertainty around B19V causation [22].

There is limited knowledge of B19V prevalence in the Australian population, which is currently limited to one study, where age specific immunity was estimated in the state of Victoria [2]. That study, conducted in the 1990s, showed the detection of IgG antibodies in 28% of children aged 0–9, increasing to 51% in the next decade of life, and again rising to 78% in those over the age of 50 [2]. The study is in concordance with previous work from Germany, reporting a rise in exposure from 20% in children (1–3 years) to 67% in adolescents (18–19 years), and further increasing to 79% in the elderly (65–69 years) [25].

There is limited understanding of current seroprevalence of antibodies to B19V in the Australian population, and therefore the population-wide immunity status. Given the potential complications arising from B19V infection during pregnancy, the incomplete understanding of B19V disease causation and the potential for B19V to be transmitted by blood transfusion and organ transplantation, there are potential implications for both public health as well as transfusion and transplantation safety in Australia. This study aimed to provide a current estimate of B19V seroprevalence in a cohort of Australian blood donors, along with a paediatric population to determine the underlying seroprevalence in Australia and therefore provide information on disease susceptibility in age cohorts.

## Methods

### Study design and population

This was cross-sectional serosurvey. A sample size of 2200 adult samples was estimated to be suitable using standard procedures [26] with the following assumptions: there is a similar rate of B19V exposure expected in this cohort to that estimated for the Victorian donor population (64%) [2], a random selection of samples, an absolute precision of 2% and a 95% confidence interval (CI). A sub-section of samples were selected from those collected for a separate research project [27], when testing was complete and where an adequate volume remained. Samples were selected (target of 324 per state/territory, totalling 2268 samples) from Queensland (11 to 16 June, 2016), New South Wales (including samples from the Australian Capital Territory; collected: 17 June to 10 September, 2016), Victoria (30 June to 1 July, 2016), Tasmania (29 June to 8 July, 2016), South Australia (29 June to 4 July, 2016), Northern Territory (9 August to 16 September, 2016), and Western Australia (19 to 27 August, 2016). Samples were age and sex stratified, with 27 male and 27 female samples targeted for each of the following age groups per state: 16–24, 25–34, 35–44, 45–54, 55–64 and ≥ 65 years. Samples (*n* = 223) from paediatric patients (0 to 15 years) in Queensland were also included (between 9 March, 2015 and 16 June, 2016).

### Sample collection

For donor samples, whole blood was collected into 6 mL dipotassium ethylenediaminetetraacetic acid (K_2_EDTA) sample preparation tubes (BD Vaccutainer ® Whole Blood Collection tube with spray-coated K_2_EDTA 6 mL, Becton Dickinson, Plymouth, United Kingdom), centrifuged at 1000 g for 10 min within 72 h of collection and stored at 4 °C temporarily. Plasma was aliquoted into Corning Cryogenic Vials (5 mL; Corning Incorporated, New York, USA) within 5 days of collection and frozen at − 30 °C prior to testing. Demographic information (age, sex and state of residence) were obtained for each sample, prior to sample de-identification and assignment of a unique study number. Paediatric serum samples were sourced from patients presenting for non-infectious investigations. Whole blood was collected and processed into serum as per Pathology Queensland standard procedures. Basic demographic information, including age and sex, accompanied the samples.

### B19V IgG serological testing

All samples were tested for the presence of B19V IgG with an enzyme-linked immunosorbent assay (ELISA) (Anti-Parvovirus B19 ELISA (IgG) Test; Euroimmun AG, Luebeck, Germany), as per the manufacturer’s instructions. This ELISA has a reported specificity (100%), sensitivity (100%) and 0% reporting of cross reactivity (including, but not limited to, against cytomegalovirus, herpes simplex virus 1, influenza A virus, influenza B virus, measles virus, mumps virus, respiratory syncytial virus, and rubella virus, as well as a parainfluenza virus pool), as determined by in house testing performed by the manufacturer. However, an independent study suggests a slightly lower sensitivity (94.3%) and specificity (92.3%) [28]. The antigen used in this assay was a recombinant VP2 protein expressed in eukaryotic cells [28]. Each plate included positive and negative controls, along with a calibrator. An automated pipetting machine (TECAN Freedom Evo; Tecan Group Ltd., Männedorf, Switzerland) was used and absorbance values read using the Synergy H1 Hybrid Multi-Mode Microplate Reader (Biotek, Vermont, USA) at 450 nm and 630 nm. A ratio of the extinction of the sample over that of the calibrator was calculated, with results interpreted as negative if < 0.8, borderline if ≥0.8 to < 1.1, and positive if ≥1.1. All samples were screened once initially, with reactive samples confirmed by a second round of testing. Samples producing discordant results were tested a third time. Samples were considered to be positive for B19V IgG if a reactive result was obtained on two separate screenings. Samples classed as ‘borderline’ were treated as if they were reactive.

### Statistical analyses

Donor/patient demographic details were recorded in a Microsoft Excel 2010 (Microsoft, Washington, USA) database. The proportion of individuals B19V IgG positive for each age group, sex and state of residencegeographical, residential location, along with overall seroprevalence estimates, with a 95% CI was calculated using standard methods [29]. National age-standardised seroprevalence estimates were calculated using relevant donor/patient prevalence data with the 2016 census data (2016 census age (in five year groups) by sex) of the Australian population aged 0–79 used as the standard population [30]. Child-bearing age was defined as those women in the age range from 16 to 44 years, as previously defined [31]. Univariate logistic regression was performed to assess associations between individual independent variables and B19V IgG seropositivity. Variables with *p*-values < 0.1 in the univariate analyses were included in a multivariate logistic regression model. IBM SPSS Statistics 23 (IBM Centre, NSW, Australia) was used for both the univariate and multivariate logistic regressions. Statistical significance was determined when *p* ≤ 0.05.

## Results

Samples from 2221 Australian blood donors and 223 Queensland paediatric samples were screened for the presence of B19V IgG (Table 1). No blood donor reported B19V infection to the Blood Service pre- or post-donation and BV19 was neither suspected nor investigated in the paediatric population. Overall, 57.90% (95% CI: 55.94%–59.85%) of samples tested positive for B19V IgG, indicating previous exposure (Table 2). The national age-standardised seroprevalence of B19V in Australian’s aged 0 to 79 years was estimated to be 54.41% (95% CI: 54.39%–54.43%).

**Table 1:** Age, sex and state breakdown of samples included in study

State	n	Age group (years)									Male (%)
		0–4	5–9	10–15	16–24	25–34	35–44	45–54	55–64	65–80	
BLOOD DONOR											
Total	2221	–	–	–	374	376	376	376	367	352	49.93%
New South Wales	323	–	–	–	54	54	54	54	54	53	49.85%
Northern Territory	293	–	–	–	53	54	54	53	49	30	51.19%
Queensland	323	–	–	–	53	54	54	54	54	54	50.15%
South Australia	321	–	–	–	54	54	54	53	52	54	49.84%
Tasmania	320	–	–	–	53	54	53	54	52	54	49.69%
Victoria	317	–	–	–	53	52	53	54	52	53	48.90%
Western Australia	324	–	–	–	54	54	54	54	54	54	50.00%
PEDIATRIC											
Total (Queensland only)	223	69	88	66	–	–	–	–	–	–	49.78%

**Table 2:** B19V seropositivity in Australian samples (blood donor and paediatric), 2016

Variable	n	B19V IgG seropositive	
		n	% (95% CI)
Female	1224	698	57.03 (54.25–59.80)
Male	1220	717	58.77 (56.01–61.53)
Overall	2444	1415	57.90 (55–94-59.85)

*B19V*: Primate erythroparvovirus 1; *CI*: Confidence interval

Of the 2221 adult blood donor samples screened, 1360 (61.23%, 95% CI: 59.21%–63.26%) tested positive for B19V IgG. Donor age (*p* < 0.001) and state of residence (*p* < 0.001) were independently assessed and identified as being associated with B19V exposure (Table 3). Consistent with other studies, the prevalence of B19V IgG seropositivity increased with increasing age among adults, from 51.34% (95% CI: 46.27%–56.40%) in the 16–24 year age group, to 78.41% (95% CI: 74.11%–82.71%) in donors aged 65–80 years. Of the 565 females of child-bearing age (16–44 years), 52.04% (95% CI: 47.92%–56.15%) had B19V IgG. The highest prevalence of B19V exposure was in donors from Tasmania (71.88%, 95% CI: 66.95%–76.80%) and the lowest in donors from the Northern Territory (52.56%, 95% CI: 46.84%–58.28%). Sex (*p* = 0.547) was not associated with the presence of B19V IgG. Both donor age group (*p* < 0.001) and state of residence (*p* < 0.001) were associated with B19V IgG positivity in the multivariate analysis; donors in the oldest age group were 3.43 times more likely to have been exposed B19V than those in the youngest age group, while donors in the southern states, including Tasmania, Victoria and South Australia, were 2.22, 1.77, and 1.46 times more likely, respectively, to be seropositive of B19V than donors residing in the Northern Territory (Table 3).

**Table 3:** B19V IgG seropositivity in Australian blood donors, 2016

Variable	Number tested	B19V IgG seropositive		Univariate logistic regression analysis		Multivariate logistic regression analysis	
		n	% (95% CI)	OR (95% CI)	*P* value	OR (95% CI)	*P* value
TOTAL	2221	1360	61.23 (59.21–63.26)	–	–	–	–
Sex					**–**		
Male	1109	686	61.86 (59.00–64.72)	a	–	–	–
Female	1112	674	60.61 (57.75–63.48)	0.95 (0.80–1.13)	0.547	–	–
Age group					< 0.001		< 0.001
16–24	374	192	51.34 (46.27–56.40)	a	–	a	–
25–34	376	221	58.78 (53.80–63.75)	1.35 (1.01–1.80)	0.041	1.36 (1.016–1.82)	0.039
35–44	376	202	53.72 (48.68–58.76)	1.10 (0.83–1.47)	0.513	1.10 (0.83–1.47)	0.503
45–54	376	235	62.50 (57.61–67.39)	1.58 (1.18–2.11)	0.002	1.59 (1.19–2.13)	0.002
55–64	367	234	63.76 (58.84–68.68)	1.67 (1.24–2.24)	0.001	1.68 (1.25–2.26)	0.001
65–80	352	276	78.41 (74.11–82.71)	3.44 (2.49–4.76)	< 0.001	3.43 (2.47–4.76)	< 0.001
State/territory					< 0.001		< 0.001
New South Wales	323	199	61.61 (56.31–66.91)	1.45 (1.05–2.0)	0.024	1.38 (1.0–1.91)	0.052
Northern Territory	293	154	52.56 (46.84–58.28)	a	**–**	a	**–**
Queensland	323	186	57.59 (52.20–62.97)	1.23 (0.89–1.69)	0.211	1.16 (0.84–1.60)	0.376
South Australia	321	202	62.93 (57.64–68.21)	1.53 (1.11–2.16)	0.009	1.46 (1.05–2.03)	0.023
Tasmania	320	230	71.88 (66.95–76.80)	2.31 (1.65–3.22)	< 0.001	2.22 (1.58–3.12)	< 0.001
Victoria	317	213	67.19 (62.02–72.36)	1.85 (1.33–2.57)	< 0.001	1.77 (1.27–2.47)	0.001
Western Australia	324	176	54.32 (48.90–59.75)	1.07 (0.78–1.48)	0.661	1.01 (0.73–1.39)	0.951

*B19V*: Primate erythroparvovirus 1; *CI*: Confidence interval; a = reference group

In the paediatric cohort, 55 samples (24.66%; 95% CI: 19.01%–30.32%) were positive for B19V IgG (Table 4). Age was associated with B19V exposure (*p* = 0.029), with increases from 17.39% (95% CI: 8.45%–26.33%) in the 0–4 year age group to 36.36% (95% CI: 24.76%–47.97%) in the 10–15 year age group. Children in this older age group were 2.71 times more likely to be B19V IgG positive than those in the youngest age group. Similarly in the adult donor cohort, there was no difference in seropositivity between the sexes (*p* = 0.261).

**Table 4:** B19V IgG seropositivity in Queensland paediatric cohort, 2016

Variable	Number tested	B19V IgG seropositive		Univariate analysis	
		n	% (95% CI)	OR (95% CI)	P value
TOTAL	223	55	24.66 (19.01–30.32)	–	–
Sex					
Male	111	31	27.93 (19.58–36.27)	a	
Female	112	24	21.43 (13.83–29.03)	1.42 (0.77–2.62)	0.261
Age group					0.029
0–4	69	12	17.39 (8.45–26.33)	a	–
5–9	88	19	21.59 (12.99–30.19)	1.31 (0.59–2.92)	0.512
10–15	66	24	36.36 (24.76–47.97)	2.71 (1.22–6.04)	0.014

*B19V*: Primate erythroparvovirus 1; *CI*: Confidence interval; a = reference group

## Discussion

This is the first, large scale epidemiological study conducted in Australia investigating the seroprevalence of B19V IgG, which will provide an understanding of the Australian-wide immunity to B19V. The overall seroprevalence of B19V in this study was 57.90%, giving an age-standardised seroprevalence of 54.41% among the Australian population aged 0–79 years. The B19V seroprevalence in the Australian blood donor population was 61.23%, which was similar to reported seroprevalence in blood donors from a number of other countries such as Spain (64.70%), Brazil (60.00%) and Japan (67.90%) [32–34]. The results in blood donors are likely applicable to the general population and therefore this study demonstrates that just under-half of the Australian population is susceptible to B19V. This has possible implications for non-immune obstetric patients, vulnerable patients at increased risk of complications such as those with sickle cell anaemia, as well as for susceptiable transfusion and transplantation recipients.

We observed considerable variation in the rate of previous exposure to B19V blood donors residing in different geographical locations in Australia [35]. Interestingly, the highest prevalence of B19V IgG seropositivity was amongst donors from Tasmania, the most southern and temperate state in Australia. The southern states of Victoria and South Australia also had a higher seroprevalence rate than those in the north of the country. This is consistent with previous studies that suggest B19V infection is more prevalent in temperate climates [1, 2]. A number of other diseases exhibit similar geographic distributions, with lower seroprevalence rates in tropical areas compared with temperate regions, and studies investigating the mechanisms behind this should be encouraged.

Age was also associated with B19V IgG seropositivity in both the paediatric and donor cohorts, with increasing age linked to higher rates of exposure. This increase in seroprevalence was anticipated and is consistent with previous studies [2, 34, 36–38]. Our results show 17.39% of Queensland children aged 0–4 years and 21.59% of those aged 5–10 years were B19V IgG positive. This estimate is slightly lower than the previously described 28% of children aged 0–9 in the Victorian study [2], but expected, given the results from this study, which suggest geographical variability in B19V seropositivity in donors with a higher a rate in Victoria. Additional studies in paediatric populations from the other states in Australia, including Victoria, would be of interest. Data comparing the 0–4 years and 10–15 year old subjects show an approximate 2% yearly risk of seroconversion.

Just over half of Australian women of child bearing age had been previously exposed to B19V, suggesting that just under half of Australian women in this category are potentially not immune to B19V and therefore remain susceptible. This is particularly of concern for these non-immune women contemplating pregnancy, as B19V may cause hydrops fetalis and other adverse manifestations during gestation [20]. Additional studies on the rate of B19V seropositivity in pregnant women would support our observation. Consistent with the geographical differences in B19V seroprevalence described above, women of child-bearing age residing in countries with a higher latitude, including Denmark, Ireland, the Netherlands and Canada (Montreal), have a higher B19V IgG prevalence, ranging from 63 to 70% [2, 39–43]; whereas lower prevalence rates were observed in women from tropical regions, including Taiwan (36%) and Singapore (23%) [44, 45]. It is not surprising that in Australia, a large country spanning tropical through to temperate climates, the overall seroprevalence rate of B19V is in the mid-range of such estimates. However, B19V IgG seropositivity is around 25% in South African women of childbearing age [46], demonstrating that factors in addition to or other than climate are likely to be important determinants of virus transmission. Given the differences in prevalence between the states observed here, further studies could investigate the level of B19V immunity in Australian women of child-bearing ages in regions of different latitude to investigate this effect further.

We did not observe a difference in B19V seroprevalence between the sexes in either blood donors or in children, which is consistent with a number of previous studies [1, 2]. However, this conflicts with a German B19V seroprevalence population study, where females were reported to have a higher rate of B19V IgG positivity when compared to males, in both adults and children [25], and also the Taiwanese population, where a similar pattern was observed, albeit lower overall [44]. This could be a reflection of different samples used in the studies or differences between assays used, or, if real, could be a reflection of different behaviours among adults between geographical localities.

Populations with lower protective immunity may represent regions with higher susceptibility to B19V infection. Our data suggest a lower B19V prevalence rate in younger Australians, along with individuals residing in the northern states of Australia. Given that B19V can be transmitted though blood transfusion [12], infection in blood donors may be asymptomatic [47], and the demonstrated increasing seroconversion rate with increasing age in these data, transfusion-transmission of B19V is a possible risk. However, symptomatic disease secondary to transfusion-transmission is only reported rarely in the literature and the benefits of blood donor screening may be minimal [12]. Moreover the likelihood of community-acquired B19V infection is far more likely in many circumstances than transfusion-transmission. It is recommended that patient groups at increased risk of infection, regardless of their transfusion status, be monitored for B19V symptoms, given the potential for treatment [12].

This study has a number of limitations. Only one, commercially available, B19V IgG ELISA was used and the serological results were based on repeat reactivity with this one assay. Confirmatory testing with a second ELISA with a different recombinant antigen would provide more confidence in the estimates. Comparing seroprevalence results between different studies using different ELISAs is known to be problematic [48], thus the comparisons discussed above should be interpreted with this in mind. Adult samples were restricted to blood donor samples and the paediatric samples to patients from Queensland only, and thus neither group may be representative of the entire general Australian population. B19V infections exhibit seasonal activity, with peaks in infections in late winter and early spring [2, 25, 49], and are believed to cycle between 2 years of epidemic activity followed by 2 years of endemicity [2] However, as this was a seroprevalence study looking at prior exposure to this virus rather than acute infection, the timing of sample collection, which was in the Australian winter and early spring of a year with unknown endemicity/epidemicity, is unlikely to impact on B19V IgG antibody status.

## Conclusions

Since the discovery of B19V in Australia in 1975, there has been no national study of its seroprevalence among the general population of Australia. This is the first comprehensive, large scale, national seroprevalence study of B19V exposure in the Australian population. The overall seroprevalence of B19V IgG in this study was 57.90%, giving an age-standardised seroprevalence of 54.41% among Australians aged 0–79. We show a clear pattern of increasing seroprevalence with increasing age and reveal higher rates of seropositivity in the southern states. Importantly, we demonstrate that just over half of the Australian population is expected to be susceptible to B19V infection if exposed, which has possible implications for the care of non-immune obstetric patients, immunosuppressed patients and those with haematological abnormalities, as well as transfusion and transplantation recipients.

## Abbreviations

B19V: Primate erythroparvovirus 1
CI: Confidence Interval
DNA: Deoxyribonucleic acid
ELISA: Enzyme-linked immunosorbent assay
IgG: Immunoglobulin G
K_2_EDTA: Dipotassium ethylenediaminetetraacetic acid
